# Transcriptome analysis to identify candidate genes associated with the yellow-leaf phenotype of a *Cymbidium* mutant generated by γ-irradiation

**DOI:** 10.1371/journal.pone.0228078

**Published:** 2020-01-29

**Authors:** Sang Hoon Kim, Se Won Kim, Gah-Hyun Lim, Jae Il Lyu, Hong-Il Choi, Yeong Deuk Jo, Si-Yong Kang, Byoung-Cheorl Kang, Jin-Baek Kim

**Affiliations:** 1 Advanced Radiation Technology Institute, Korea Atomic Energy Research Institute, Jeongeup, Republic of Korea; 2 National Institute of Agricultural Sciences, Rural Development Administration, Jeonju, Republic of Korea; 3 Department of Plant Science, Plant Genomics and Breeding Institute, and Vegetable Breeding Research Center, College of Agriculture and Life Sciences, Seoul National University, Seoul, Republic of Korea; Nigde Omer Halisdemir University, TURKEY

## Abstract

Leaf color is an important agronomic trait in flowering plants, including orchids. However, factors underlying leaf phenotypes in plants remain largely unclear. A mutant displaying yellow leaves was obtained by the γ-ray-based mutagenesis of a *Cymbidium* orchid and characterized using RNA sequencing. A total of 144,918 unigenes obtained from over 25 million reads were assigned to 22 metabolic pathways in the Kyoto Encyclopedia of Genes and Genomes database. In addition, gene ontology was used to classify the predicted functions of transcripts into 73 functional groups. The RNA sequencing analysis identified 2,267 differentially expressed genes between wild-type and mutant *Cymbidium* sp. Genes involved in the chlorophyll biosynthesis and degradation, as well as ion transport, were identified and assayed for their expression levels in wild-type and mutant plants using quantitative real-time profiling. No critical expression changes were detected in genes involved in chlorophyll biosynthesis. In contrast, seven genes involved in ion transport, including two metal ion transporters, were down-regulated, and chlorophyllase 2, associated with chlorophyll degradation, was up-regulated. Together, these results suggest that alterations in chlorophyll metabolism and/or ion transport might contribute to leaf color in *Cymbidium* orchids.

## Introduction

Orchids such as *Cymbidium*, *Dendrobium*, *Oncidium*, and *Phalaenopsis* are important cash crops worldwide, and the orchid industry has contributed substantially to the economy of many Southeast Asian countries [1, 2]. Because of its fragrant flowers and straight leaves, *Cymbidium* is a popular orchid in China, Korea, and Japan [3–5]. In addition to its floral diversity, the color and pattern of *Cymbidium* leaves is an important marketable feature [6–8] and the focus of *Cymbidium* breeding programs [9].

Although the biological significance and bio-diversity of genome sizes in angiosperms have received considerable attention in recent years [10, 11], the genomic organization of Orchidaceae remains poorly characterized. This could be largely owing to the poor genome representation of Orchidaceae, which contains over 28,000 species distributed in 763 genera [12]. Thus far, the genomes of four orchids, *Phalaenopsis equestris*, *Dendrobium catenatum*, *Dendrobium officinale*, and *Apostaceae shengen*, have been sequenced [13]. The genome sequences revealed that genome sizes in Orchidaceae have a 168-fold, making them the most diverse among angiosperms [14]. The assembled sequenced genomes of orchids have predicted gene numbers ranging from 28,910 in *P*. *equestris* to 35,567 in *D*. *officinale* [15, 16].

Recent advances in orchid genome research have facilitated conventional and mutagenesis-based breeding approaches designed to generate varieties with unique flower and leaf phenotypes [17]. Leaf yellowing is generally associated with chlorophyll (Chl) biosynthesis or degradation pathways, both of which are mediated by multiple enzymatic steps. Thus, a block in any of the step leading to Chl synthesis can potentially result in low Chl content and thereby altered leaf color [18, 19]. The leaf color phenotype has been extensively studied in rice and has provided insights into the steps involved in Chl biosynthesis and degradation, chloroplast developments, tetrapyrrole synthesis, and photosynthesis [20]. These studies have led to the isolation of diverse leaf colors and patterns including albino, light and purple green leaves, as well as striped and zebra-patterned leaves [21]. Thus far, over 50 genes contributing to leaf color have been characterized in rice and 13 of these function in Chl biosynthesis [22–27]. These studies suggest that genes regulating leaf color can, directly or indirectly, contribute to Chl biosynthesis and/or structure of the chloroplast and that Chl and anthocyanin content are major contributors to the leaf color. The biosynthesis of Chl is also dependent on biochemical processes that regulate uptake and transport of macronutrients including metals and cofactors. The uptake, chelation, trafficking, and storage of metal ions is tightly regulated to maintain an optimal intracellular concentration of metal ions for Chl biosynthesis. Metal ions also play an essential roles in photosynthetic and metabolic processes associated with leaf color [28–31]. For example, a mutation in iron-regulated transporter 1 alters composition and abundance of the photosynthetic apparatus in Arabidopsis and causes drastic reduction in growth rate and fertility [32, 33]. In this study, we used the γ-ray-based mutagenetic procedure to isolate a leaf-color mutant in *Cymbidium*, and we show that the mutant’s yellow leaf-color is likely associated with Chl degradation and/or ion transport.

## Materials and methods

### Plant materials

The wild-type (WT) *Cymbidium* hybrid, RB003 was derived from a cross between *C*. *sinense* × *C*. *goeringii*. A yellow leaf-color mutant, designated as S12, was derived from RB003 by ^©^-ray mutagenesis, which was carried out at the Korea Atomic Energy Research Institute. All plants were grown in a greenhouse under natural light and photoperiod.

### RNA extraction

Extraction of total RNA from six-month old leaves of the WT and the S12 mutants was carried out using a RNease Plant Mini kit (Qiagen, Hilden, Germany), following the manufacturer’s instructions. RNA quality and concentration were determined using on a Nanodrop 2000 spectrophotometer (Thermo Fisher Scientific, Waltham, MA, USA).

### Quantitative real-time PCR (qRT-PCR) analysis

Reverse transcription (RT) and first-strand cDNA synthesis were carried out using a ReverTra Ace-α- kit (Toyobo Co. Ltd, Osaka, Japan). Quantitative RT-PCR was carried out with iQ SYBR Green Supermix (Bio-Rad, Hercules, CA, USA). Quantitative PCR was performed using CFX96 Touch Real-Time PCR Detection System (Bio-Rad, Hercules, USA). The PCR conditions included and initial denaturation step of 95°C for 10 min followed by 35 cycles of 95°C for 15 s, 56–62°C for 15 s and 72°C for 30 s. Each sample was run in triplicates and *ACTIN* was used as an internal control. Cycle threshold values were calculated using Bio-Rad CFX Manager 3.1 software (Bio-Rad, Hercules, USA). Gene-specific primers used for quantitative RT-PCR are described in S1 Table.

### Chl and carotenoid content assay

Six-month-old leaves of the WT and the S12 mutants were sampled for Chl and carotenoid determination. Amounts of Chls *a* and *b* and carotenoids were estimated following the method of Lichtenthaler [34]. The fresh leaf samples were ground using liquid nitrogen and suspended in 96% ethanol (Sigma, St. Louis, MO, USA). This extract was vortexed and placed at room temperature in dark for 24 h. The absorption spectra of the extract was measured at 470, 648.6, and 664.2 nm using a UV-1800 spectrophotometer (Shimadzu, Kyoto, Japan).

### RNA sequencing and *de novo* assembly

The cDNA libraries were prepared from both the WT and the S12 leaves. Raw reads were trimmed by filtering out adaptor-only nucleotides that were smaller than 75 bp using Trimmomatic (v0.32) [35]. *De novo* assembler Trinity (v2.2.0) was used to construct large contigs from the filtered reads [36]. Trinity is a representative RNA assembler based on the de Bruijn graph algorithm. The assembly pipeline of Trinity consists of three consecutive modules: Inchworm, Chrysalis, and Butterfly. Protein coding sequences (CDSs) were extracted from the reconstructed transcripts using TransDecoder (v3.0.1), a utility included with Trinity to assist with the identification of potential coding region [37]. The prediction of coding regions is based on search of all possible CDSs, verification of the predicted CDSs by GENEID [38], and selecting the region that has the highest score among candidate sequences.

### Functional annotation

Trimmed read were annotated with protein databases including gene ontology (GO), database for annotation, visualization, and integrated discovery (DAVID) [39], Kyoto Encyclopedia of Genes and Genomes (KEGG), and eukaryotic clusters of orthologous genes (KOG) databases. GO includes biological processes (BP), cellular component (CC), and molecular function (MF). KEGG is a major recognized pathway-related database that integrates genomic, biochemical, and systemic information. DAVID is a web-accessible program that integrates functional genomic annotations with intuitive graphical displays, which highlights pathway members within the biochemical pathways provided by KEGG. KOG categories were obtained via comparisons to the KOG database using RPS-BLAST (included with BLAST v2.2.26) [40].

### Identification of differentially expressed genes between wild-type and S12 mutant

Gene expression profiles were analyzed using the method of RNA-Seq following Expectation Maximization (RSEM) [41]. The unique feature of RSEM is that it does not rely on a reference genome. RSEM uses the Bowtie alignment program to align transcripts. Differentially expressed genes (DEGs) were identified using edgeR [42], a Bioconductor package based one the generalized linear model that analyzes RNA-Seq data by considering gene expression as a negative binomial. We used a false discovery rate (FDR) < 0.05 significance cut-off for multiple testing adjustments [43].

## Results

### Reduced accumulation of Chls and carotenoids in the S12 mutant

**Fig 1:**
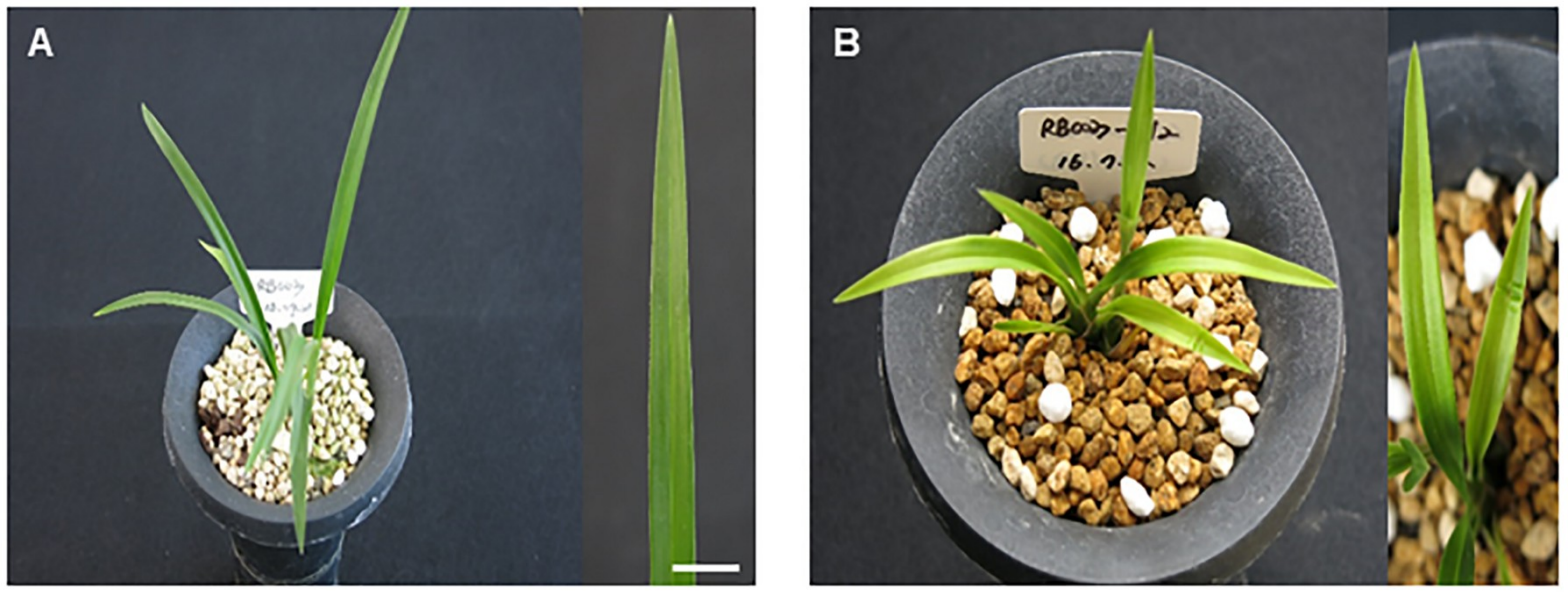
Morphological phenotypes of typical wild-type and S12 mutant plants of *Cymbidium* hybrid RB003. (A) Wild-type plants (B) The yellow leaved S12 mutant.

**Fig 2:**
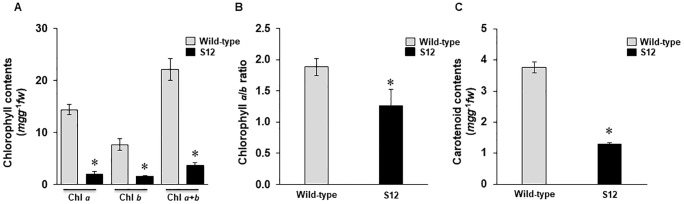
Relative levels of chlorophylls *a* and *b* and carotenoids in the wild-type and S12 mutant. (**A**) Total chlorophyll content, (**B**) chlorophyll *a*/*b* ratio, and (**C**) carotenoid contents of wild-type and S12 mutant leaves. Error bars represent standard deviation (*n* = 3). The experiment was repeated three times with similar results.

The S12 mutant developed yellow-colored leaves from the seedling stage to maturity (Fig 1). To determine if this was associated with reduced Chl or carotenoid content, we quantified Chl *a* and Chl *b* as well as total carotenoids. A significant reduction in Chl and carotenoid contents was observed in the S12 mutant leaves; Chl *a*, Chl *b*, and carotenoid levels were reduced by 85%, 78%, and 65%, respectively (Fig 2A and 2C). The ratio between Chl *a* and Chl *b* also differed nominally but significant difference between the WT and the S12 mutants. The Chl *a*/*b* ratio serves as a useful indicator of plant response to shading [44] (Fig 2B).

**Fig 3:**
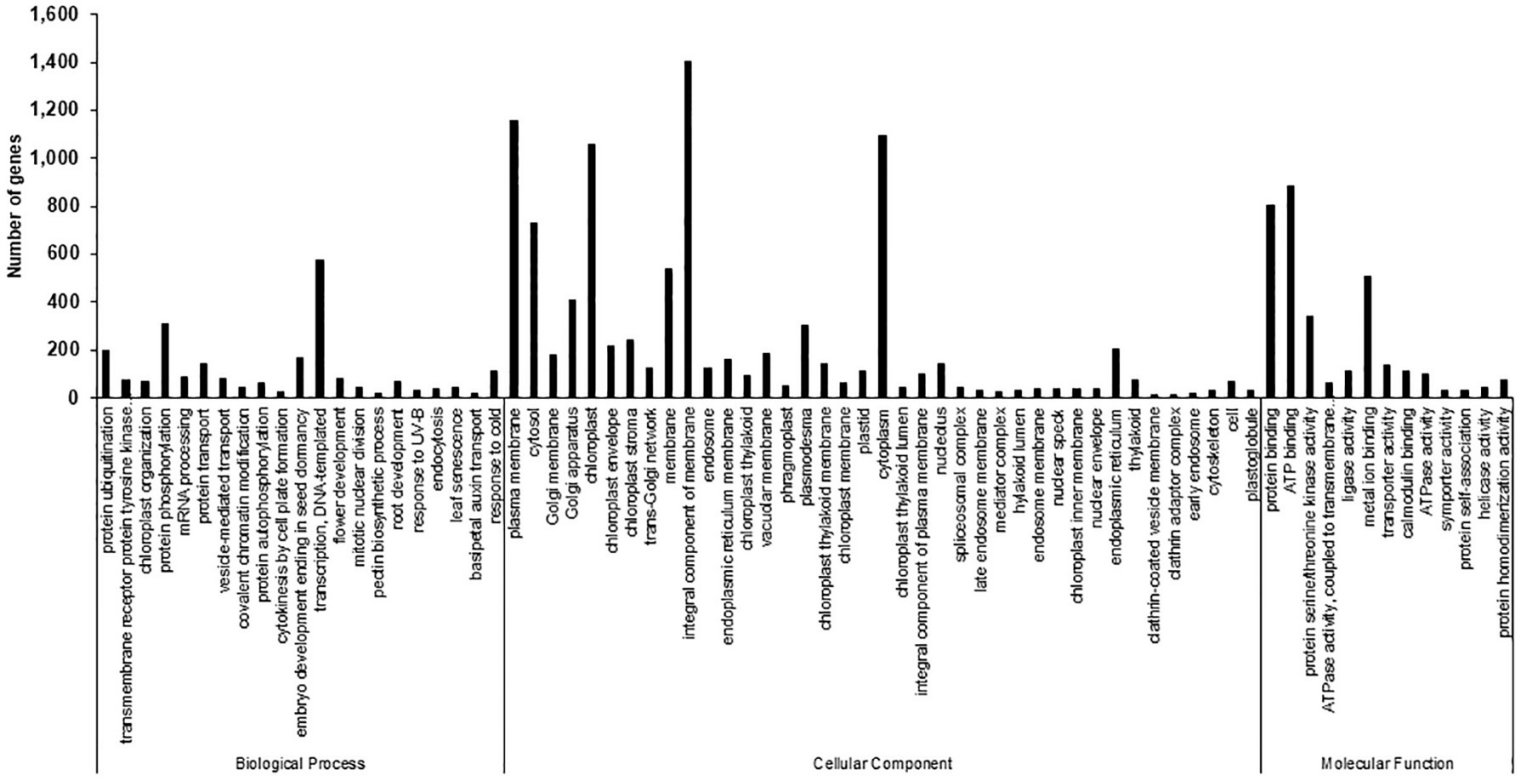
Distribution of annotated sequences based on gene ontology (GO) analysis. GO functional classification assigned 144,918 unigenes to 73 subcategories under the three main GO categories of biological process, cellular component, and molecular function. The *x*-axis indicates the subcategories, and the *y*-axis indicates the number of genes in each category.

To determine molecular changes underlying the S12 mutation, we assayed changes in genome-wide transcript levels using RNA-Seq. To this end, equal amount of RNAs from the WT and the S12 mutants were used to construct cDNA libraries, and then sequenced by Trimmomatic (v0.32) platform. The average length of clean reads was 308–315 bp and a total of 30.83 million (99.92%) and 27.45 million (99.94%) clean reads were generated from the WT and the S12 mutant, respectively. A total of 225,694 transcripts were assembled into 144,918 unigenes with an average length of 1,257 bp (Table 1). We used gene ontology (GO) classification to classify the predicted functions of unigenes, which were categorized into 73 functional groups (FDR < 0.05). GO assignments were divided into three categories including biological process (BP), cellular component (CC), and molecular function (MF). Integral component of membrane (15.83%), plasma membrane (13.07%), and cytoplasm (12.36%) were dominant groups in the CC category followed by chloroplast (11.93%), cytosol (8.21%), and membrane (6.07%). Predicted proteins assigned to BP category were mainly associated with transcription (6.52%), protein phosphorylation (3.51%), protein ubiquitination (2.21%), and embryo development (1.87%). In the MF category, the most heavily represented groups were linked to ATP binding (9.99%), protein binding (9.09%), metal ion binding (5.74%), and protein serine/threonine kinase activity (3.84%) (Fig 3).

**Table 1:** Summary of RNA sequencing and *de novo* transcriptome assembly results.

	Wild-type	S12 mutant
Number of raw reads	30,860,529	27,468,828
Average length of raw reads (bp)	101	101
Number of trimmed reads	30,836,504	27,452,350
Number of trinity transcripts	225,694	
Number of unigenes	144,918	
Contig N50 (bp)	1,257	
Number of DEGs with significant expression differences (FDR < 0.05)	2,267	

**Fig 4:**
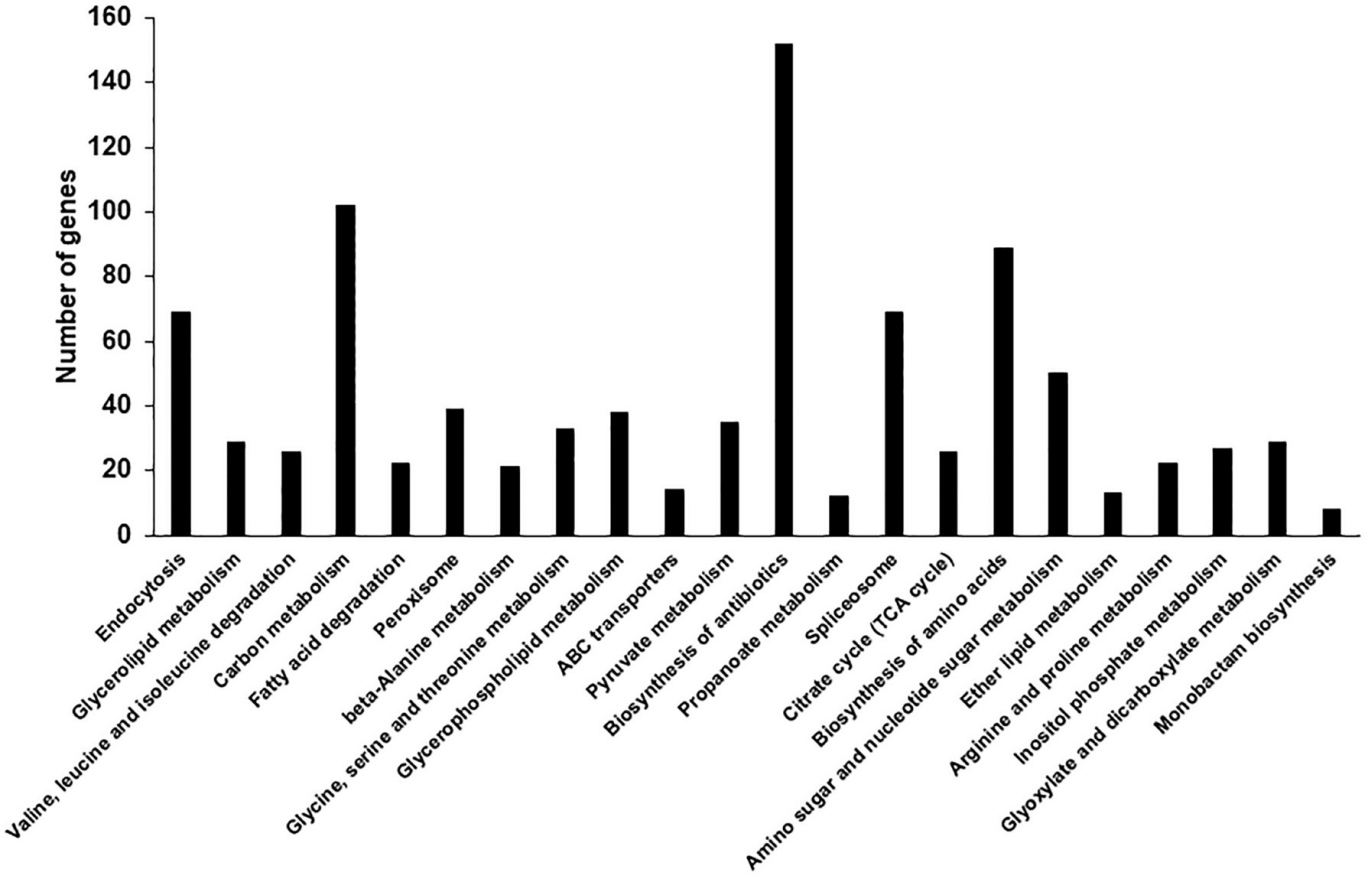
Distribution of annotated sequences based on Kyoto Encyclopedia of Genes and Genomes (KEGG) pathway analysis. The *x*-axis indicates enriched KEGG pathways, and the *y*-axis represents the number of genes within each KEGG pathway.

Next, we mapped the assembled unigenes to the reference canonical pathway in the KEGG, including metabolism, genetic information processing, environmental information processing, and cellular processes (http://www.kegg.jp/kegg/pathway.html). The 144,918 unigenes were assigned to 22 KEGG sub-pathways (Fig 4). Theses pathways included KEGG orthology (KO) entries for metabolism (734 KOs), genetic information processing (69 KOs), environmental information processing (14 KOs), and cellular processes (108 KOs) (Table 2).

**Table 2:** Functional categorization of assembled unigenes in KEGG pathways.

KEGG sub-pathways	Count
**Metabolism**	
**1.0 Global and overview maps**	**343**
01130 Biosynthesis of antibiotics	152
01200 Carbon metabolism	102
01230 Biosynthesis of amino acids	89
**1.1 Carbohydrate metabolism**	**179**
00020 Citrate cycle (TCA cycle)	26
00520 Amino sugar and nucleotide sugar metabolism	50
00620 Pyruvate metabolism	35
00630 Glyoxylate and dicarboxylate metabolism	29
00640 Propanoate metabolism	12
00562 Inositol phosphate metabolism	27
**1.3 Lipid metabolism**	**102**
00071 Fatty acid degradation	22
00561 Glycerolipid metabolism	29
00564 Glycerophospholipid metabolism	38
00565 Ether lipid metabolism	13
**1.5 Amino acid metabolism**	**81**
00260 Glycine, serine and threonine metabolism	33
00280 Valine, leucine and isoleucine degradation	26
00330 Arginine and proline metabolism	22
**1.6 Metabolism of other amino acids**	**21**
00410 Beta-alanine metabolism	21
**1.10 Biosynthesis of other secondary metabolites**	**8**
00261 Monobactam biosynthesis	8
**Genetic Information Processing**	
**2.1 Transcription**	**69**
03040 Spliceosome	69
**Environmental Information Processing**	
**3.1 Membrane transport**	**14**
02010 ABC transporters	14
**Cellular Processes**	
**4.1 Transport and catabolism**	**108**
04144 Endocytosis	69
04146 Peroxisome	39

### The implicated role of ion transport and Chl catabolism in the S12 phenotype according to DEG analysis

**Fig 5:**
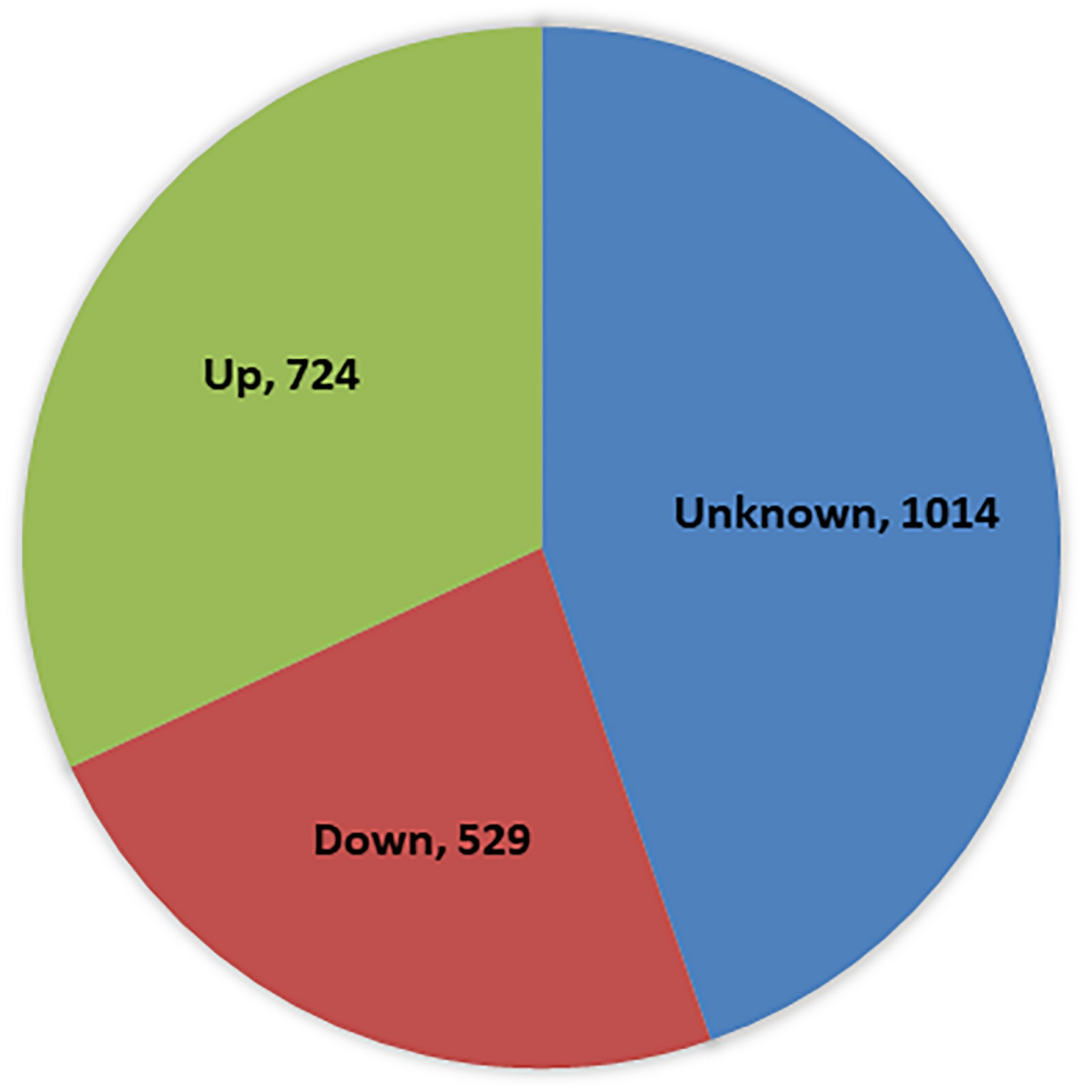
Venn diagram showing numbers of genes with altered expressions in the S12 mutant compared with wild-type. Genes were categorized on the basis of their biological functions. Genes not showing homology to genes of any known function are listed as unknown.

**Fig 6:**
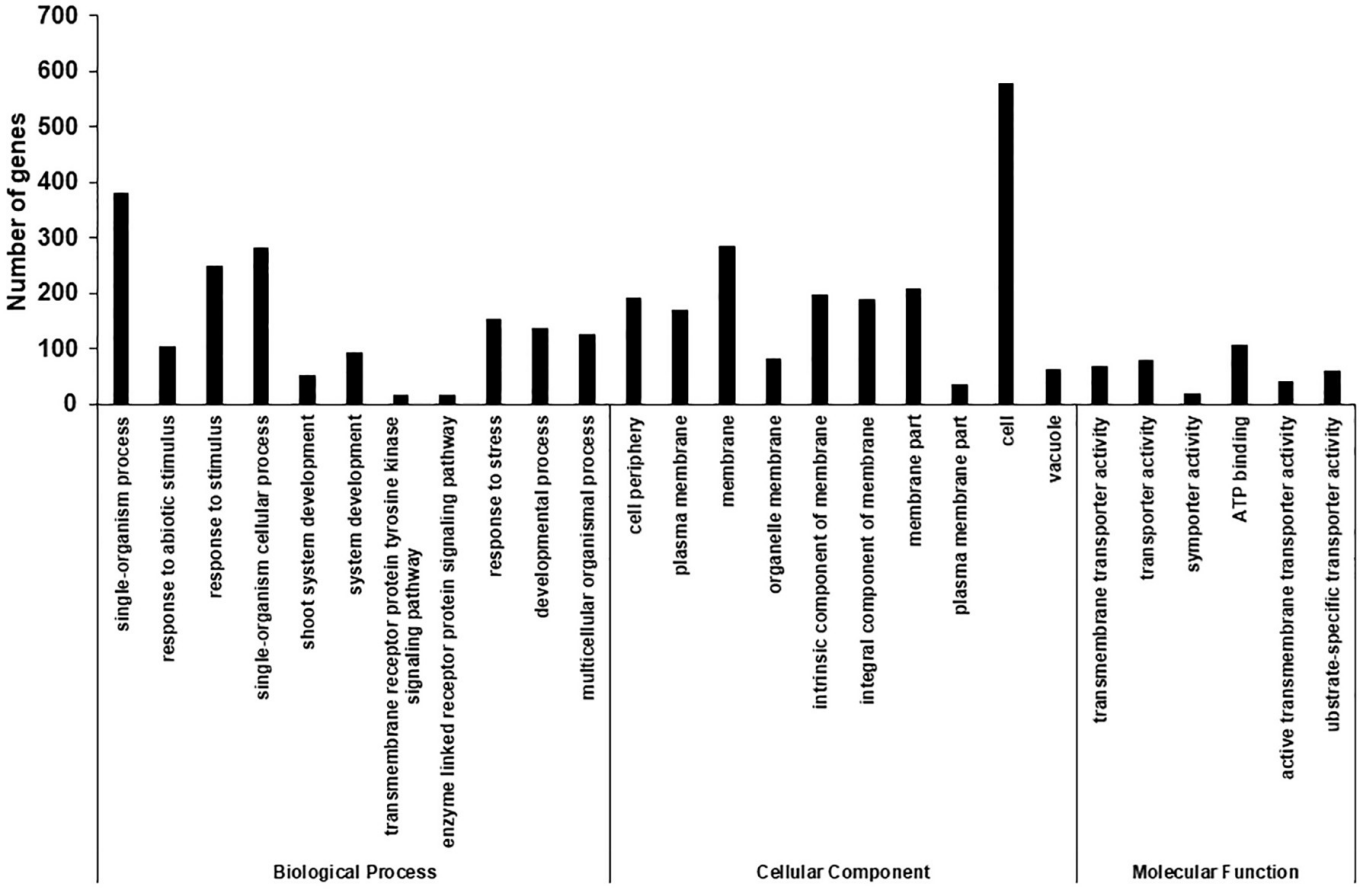
Gene ontology (GO) functional classification of differentially expressed genes (DEGs). GO classification assigned 2,267 DEGs to 27 functional groups based on their biological functions.

Gene expression analysis identified a total of 2,267 DEGs (FDR < 0.05) between the WT and the S12 mutant. Among these genes, 724 genes were up-regulated and 529 were down-regulated (Fig 5). The DEGs were categorized into 27 functional groups in GO classification (FDR < 0.05). Predicted proteins assigned to biological process (BP) were mainly associated with single-organism process, which corresponded to the largest group. Cell and membrane terms were dominant among cellular components (CC). Those assigned to molecular function (MF) were mainly linked to ATP binding and transport activity (Fig 6). Especially, when DEG GO and total GO were compared in CC and MF, percentage of membrane group and transporter activity group were higher in DEG GO.

**Fig 7:**
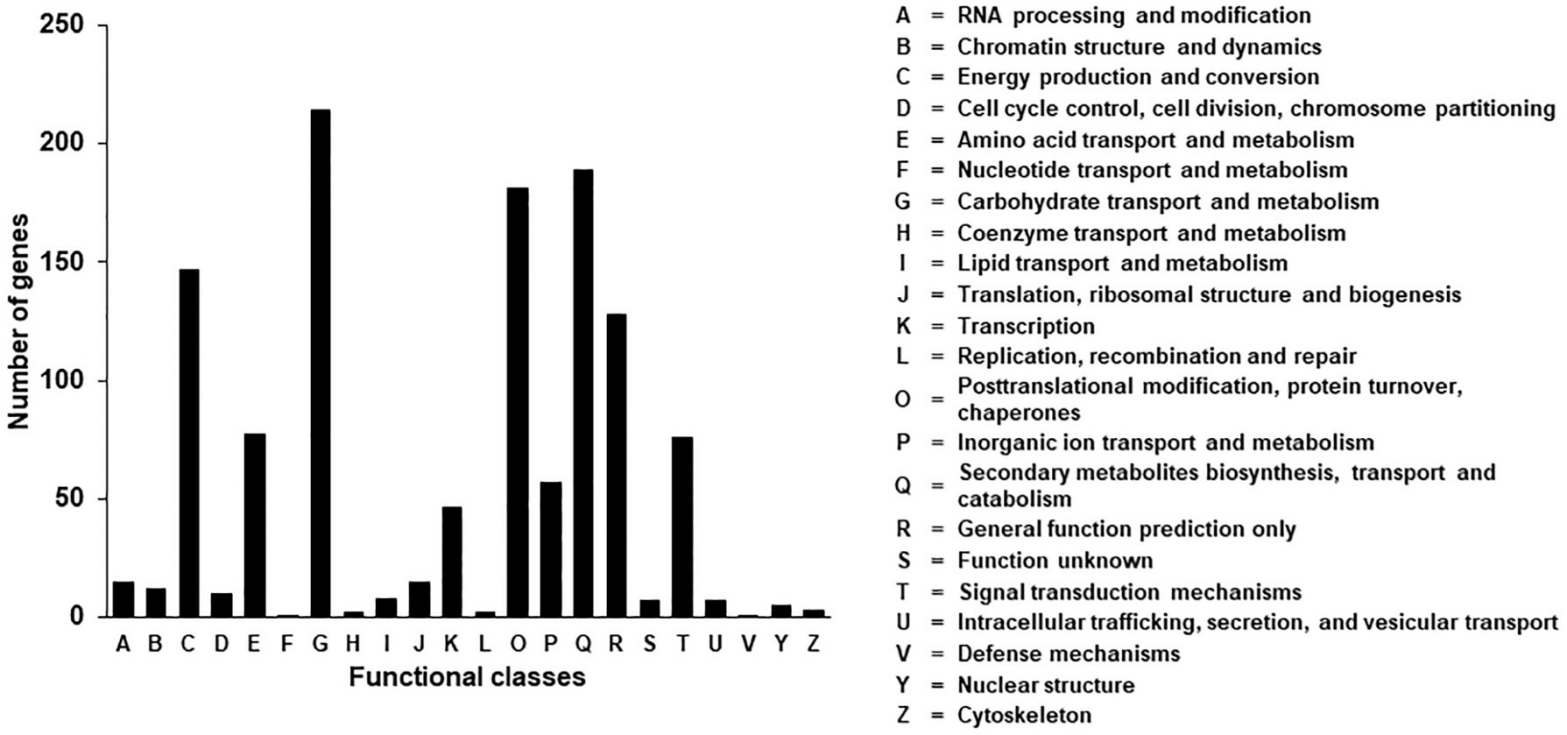
Eukaryotic clusters of orthologous genes (KOG) annotations of differentially expressed genes (DEGs). The 2,267 DEGs were aligned with the KOG database and classified into 22 molecular families. Letters on the *x*-axis refer to categories on the right. The *y*-axis indicates the number of DEGs in the corresponding KOG category.

**Fig 8:**
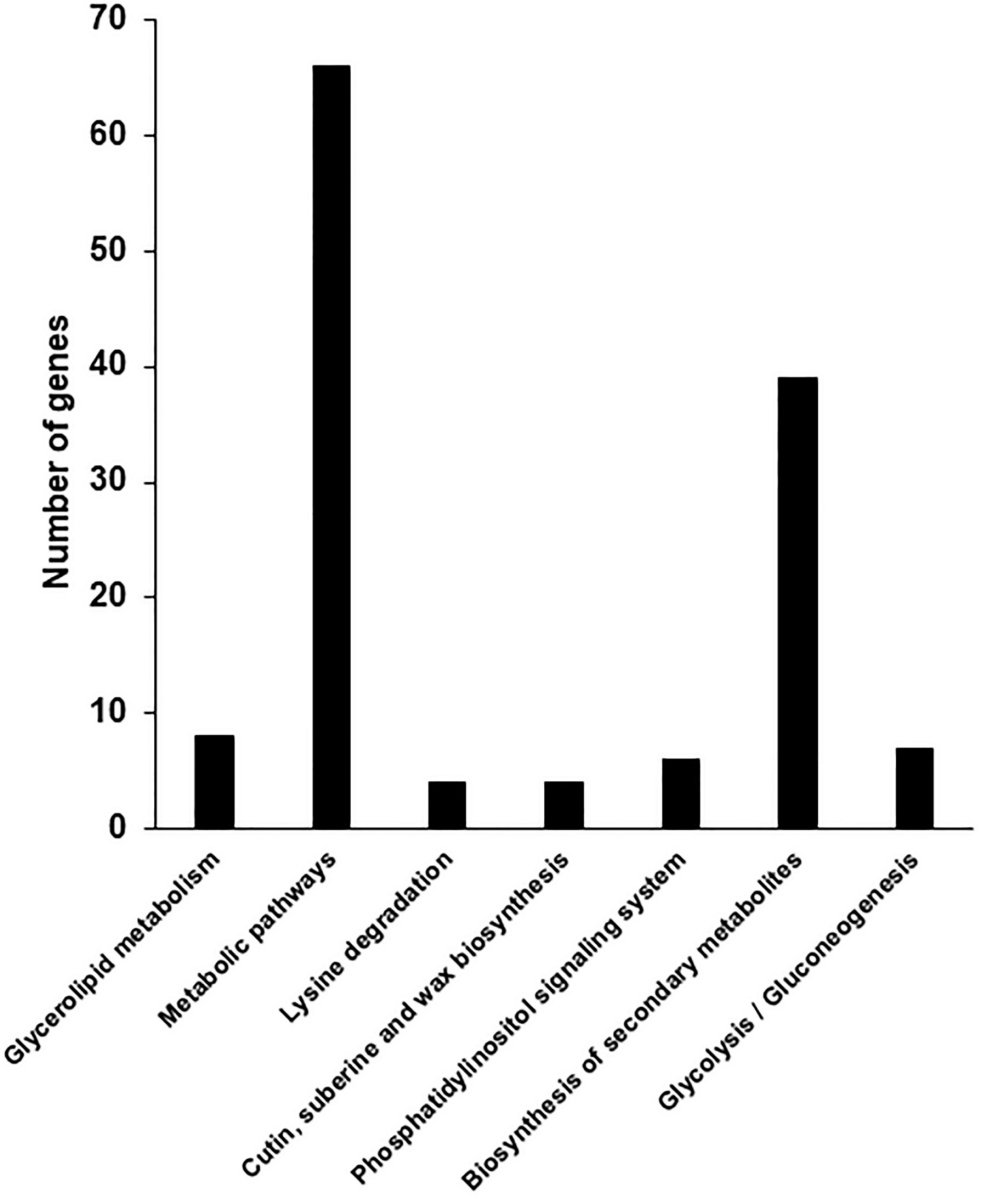
KEGG pathway representation of differentially expressed genes (DEGs) in the S12 mutant.

The DEGs were further classified into 22 functional categories using the KOG system. The largest KOG cluster was carbohydrate transport and metabolism (G), followed by secondary metabolites biosynthesis, transport and catabolism (Q), post-translational modification (O), energy production and conversion (C), and general function (R) (Fig 7). The KOG categories were further divided into multiple classes including cellular processes and signaling (273), information storage and processing (90), metabolism (705), and poorly characterized (135) classes (S2 Table). Next, we analyzed gene pathway assignment of DEGs to KEGG pathway. As a result, DEGs were assigned to seven sub-pathways (Fig 8), which in turn served as a template for assaying specific metabolic processes associated with the S12 mutant. To understand the biological roles of the screened major targets, we used DAVID to perform the GO biological process enrichment analysis. The DAVID, the most widely used on-line tool for functional classification, relies on a partitioning approach that groups genes together on the basis of their functional similarities. As predicted, a major category of DEGs was involved in membrane and chloroplast functions (Table 3). These DEGs included ABC transporters that were up-regulated in the S12 mutant and genes associated with ion transport were down-regulated (Tables 4 and 5). Interestingly, the metal ion transporters have been associated with leaf color and photosynthesis [45, 46]; their encoding genes were thus considered as candidates for the genes responsible for the phenotype seen in the S12 mutant.

**Table 3:** Functional annotation of differentially expressed genes based on DAVID.

Database		Count	*P*-value
**Cluster 1**	**Enrichment score: 7.43**		
UP_KEYWORDS	Membrane	232	1.00 × 10^−13^
UP_KEYWORDS	Transmembrane helix	185	3.50 × 10^−8^
UP_KEYWORDS	Transmembrane	185	5.30 × 10^−8^
GOTERM_CC_DIRECT	Integral component of membrane	177	9.70 × 10^−6^
UP_SEQ_FEATURE	Transmembrane region	89	3.90 × 10^−5^
**Cluster 2**	**Enrichment score: 3.91**		
UP_KEYWORDS	Nucleus	132	9.60 × 10^−10^
UP_KEYWORDS	Transcription regulation	79	9.50 × 10^−7^
UP_KEYWORDS	Transcription	80	1.20 × 10^−6^
UP_KEYWORDS	DNA-binding	70	1.30 × 10^−5^
GOTERM_MF_DIRECT	Sequence-specific DNA binding	35	1.80 × 10^−3^
GOTERM_MF_DIRECT	Transcription factor activity, sequence-specific DNA binding	69	6.50 × 10^−3^
GOTERM_BP_DIRECT	Transcription, DNA-templated	75	7.40 × 10^−3^
GOTERM_BP_DIRECT	Regulation of transcription, DNA-templated	76	5.80 × 10^−2^
GOTERM_MF_DIRECT	DNA binding	71	1.00 × 10^−1^
**Cluster 3**	**Enrichment Score: 3.8**		
UP_KEYWORDS	Chloroplast	64	1.30 × 10^−6^
UP_KEYWORDS	Plastid	64	1.50 × 10^−6^
UP_KEYWORDS	Transit peptide	73	2.90 × 10^−6^
GOTERM_CC_DIRECT	Chloroplast	109	5.20 × 10^−2^
UP_SEQ_FEATURE	Transit peptide:chloroplast	25	3.50 × 10^−1^

**Table 4:** Differentially expressed genes with increased expression levels that likely lead to higher enzymatic activities.

Trinity ID	Uniprot accession	Gene symbol	Gene	logFC	FDR
TRINITY_DN71117_c0_g1	Q9SYI2	AB3B	ABC transporter B family member 3	1.028443	0.008577
TRINITY_DN85004_c0_g1	Q9LK64	AB3C	ABC transporter C family member 3	1.787229	0.013526
TRINITY_DN87313_c1_g1	Q8LGU1	AB8C	ABC transporter C family member 8	2.204471	8.69 × 10^−9^
TRINITY_DN81240_c2_g1	Q9M1C7	AB9C	ABC transporter C family member 9	2.136759	5.38 × 10^−11^
TRINITY_DN86578_c4_g1	Q9SKX0	AB13C	ABC transporter C family member 13	1.091064	0.0379
TRINITY_DN63855_c0_g2	Q9FF46	AB28G	ABC transporter G family member 28	3.192968	8.98 × 10^−5^
TRINITY_DN84784_c2_g1	Q8GU89	AB37G	ABC transporter G family member 37	1.53448	0.020845
TRINITY_DN85702_c1_g3	Q8GU88	AB39G	ABC transporter G family member 39	1.097948	0.00171
TRINITY_DN75921_c1_g1	Q94AH3	NIPA4	Magnesium transporter NIPA4	1.313692	0.00298
TRINITY_DN76502_c0_g1	Q8GYH8	SUTR42	Sulfate transporter 4;2	2.36809	1.72 × 10^−17^
TRINITY_DN85119_c0_g1	Q9LHN7	PUT4	Amino acid permease family protein	0.856779	0.038716
TRINITY_DN81780_c0_g1	Q9SQZ0	CAT7	Cationic amino acid transporter 7	2.018167	8 × 10^−5^
TRINITY_DN75271_c0_g1	Q10Q65	NRAM2	Metal transporter Nramp2	2.881755	7.46 × 10^−19^
TRINITY_DN74882_c1_g1	Q9M7I7	CLH2	Chlorophyllase-2	1.490092	0.0010
TRINITY_DN78535_c3_g1	Q84ST4	NOL	Chlorophyll(ide) b reductase	0.400089	0.5953
TRINITY_DN77744_c2_g1	Q9FYC2	PAO	Pheophorbide a oxygenase	0.534353	0.6442
TRINITY_DN81301_c1_g1	Q9MTQ6	RCCR	Red chlorophyll catabolite reductase	0.114782	0.9641

**Table 5:** Differentially expressed genes with decreased expression levels that likely lead to lower enzymatic activities.

Trinity ID	Uniprot accession	Gene symbol	Gene	logFC	FDR
TRINITY_DN74817_c0_g1	Q8L4S2	MRS2F	Magnesium transporter MRS2-F	−2.53315	0.001367
TRINITY_DN80302_c2_g1	Q058N4	MRS2B	Magnesium transporter MRS2-11	−1.10454	0.014623
TRINITY_DN35346_c0_g1	Q5JK32	HAK5	Potassium transporter 5	−7.80167	1.45 × 10^−11^
TRINITY_DN86452_c2_g1	Q67UC7	HAK17	Potassium transporter 17	−2.28674	0.000628
TRINITY_DN82926_c0_g1	O82089	CCH	Copper transport protein CCH	−2.84536	0.009691
TRINITY_DN82149_c4_g1	Q94LW6	SUT35	Sulfate transporter 3;5	−1.33172	0.018302
TRINITY_DN86390_c5_g1	Q8VZ80	PLT5	Polyol transporter	−1.74481	0.010401
TRINITY_DN81484_c2_g1	Q93ZF5	PHO11	Phosphate transporter PHO1 homolog 1	−11.8706	1.8 × 10^−17^
TRINITY_DN82844_c0_g1	Q9C9Z1	ZTP50	Zinc transporter 50	−1.51218	8.95 × 10^−5^
TRINITY_DN85192_c3_g1	Q9M1P7	BOR2	Boron transporter 2	−10.5916	2.93 × 10^−8^
TRINITY_DN55717_c0_g1	Q84KJ6	AMT31	Ammonium transporter 3 member 1	−7.6494	2.67 × 10^−7^
TRINITY_DN83319_c2_g1	Q9LS46	ALMT9	Aluminum-activated malate transporter 9	−11.5069	0.032332
TRINITY_DN82053_c3_g1	Q7XUJ2	YSL9	Metal-nicotianamine transporter YSL9	−2.88577	7.76 × 10^−13^
TRINITY_DN59808_c0_g2	P27489	CAB13	Chlorophyll a-b binding protein 13	−3.77677	0.006663
TRINITY_DN74933_c1_g1	Q9SW18	CHLM	Magnesium protoporphyrin IX methyltransferase	−1.00694	0.382536
TRINITY_DN83083_c8_g2	Q9M591	CRD1	Magnesium-protoporphyrin IX monomethylester cyclase	−0.46194	0.787932
TRINITY_DN76234_c0_g1	Q5W6H5	CHLG	Chlorophyll synthase	−1.10377	0.092632
TRINITY_DN80530_c2_g1	Q41249	PORA	Protochlorophyllide a reductase	−4.57080	0.491924
TRINITY_DN80260_c3_g15	Q5N800	NYC1	Probable chlorophyll(ide) b reductase	−2.70738	0.002168

**Fig 9:**
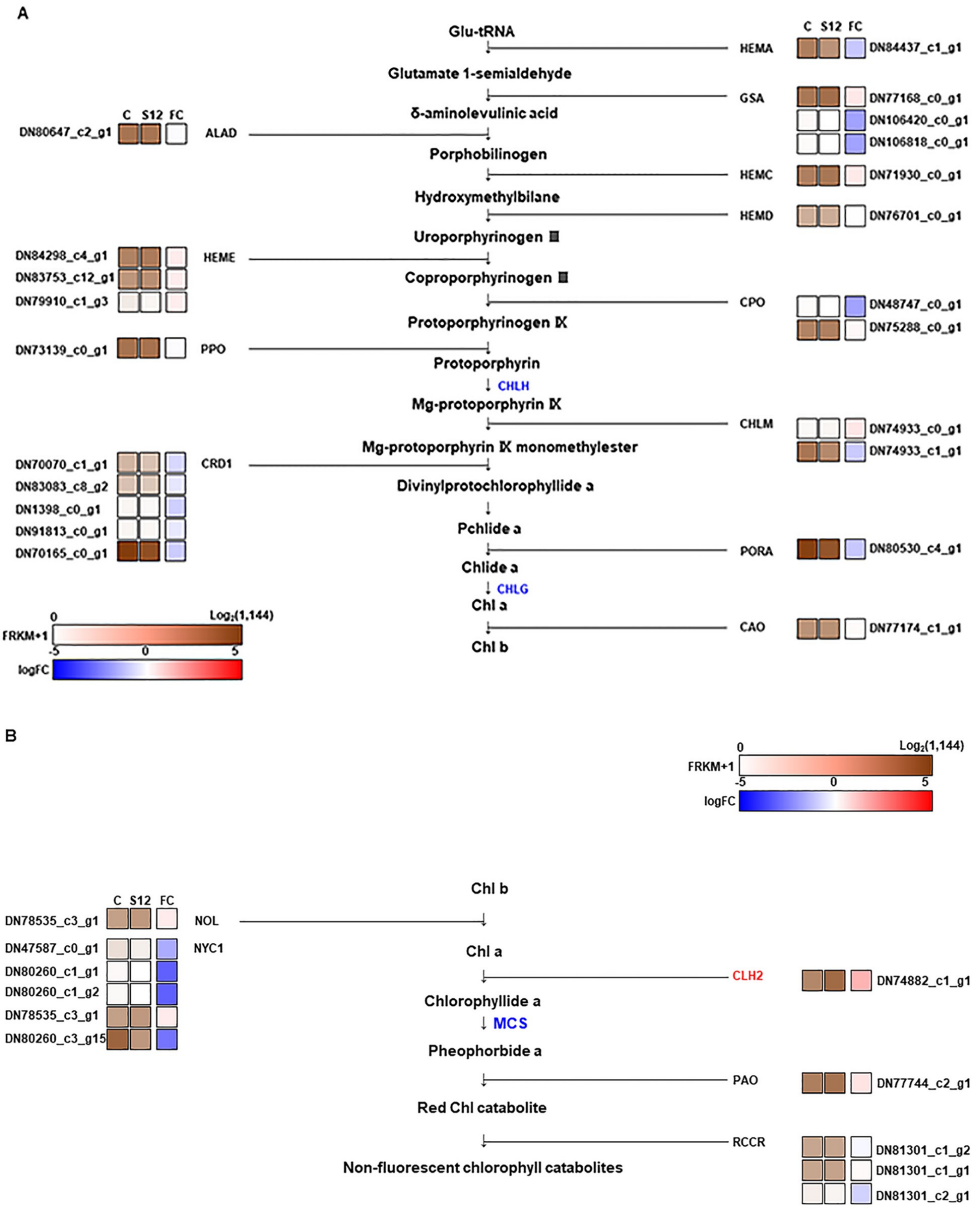
Schematic diagram of reads mapped to genes encoding proteins involved in chlorophyll biosynthesis and degradation. (**A**) Chlorophyll biosynthetic pathway. (**B**) Chlorophyll degradation pathway. C, S12, and FC stand for wild-type, S12 mutant, and fold change, respectively. In both (**A**) and (**B**), the logarithm of the FPKM (fragments per kilobase of exon per million fragments mapped) +1 value of each sample and the logarithm of the ratio of the FPKM+1 values of two samples are represented by the color scales in the bottom right-hand corner of the figure.

**Fig 10:**
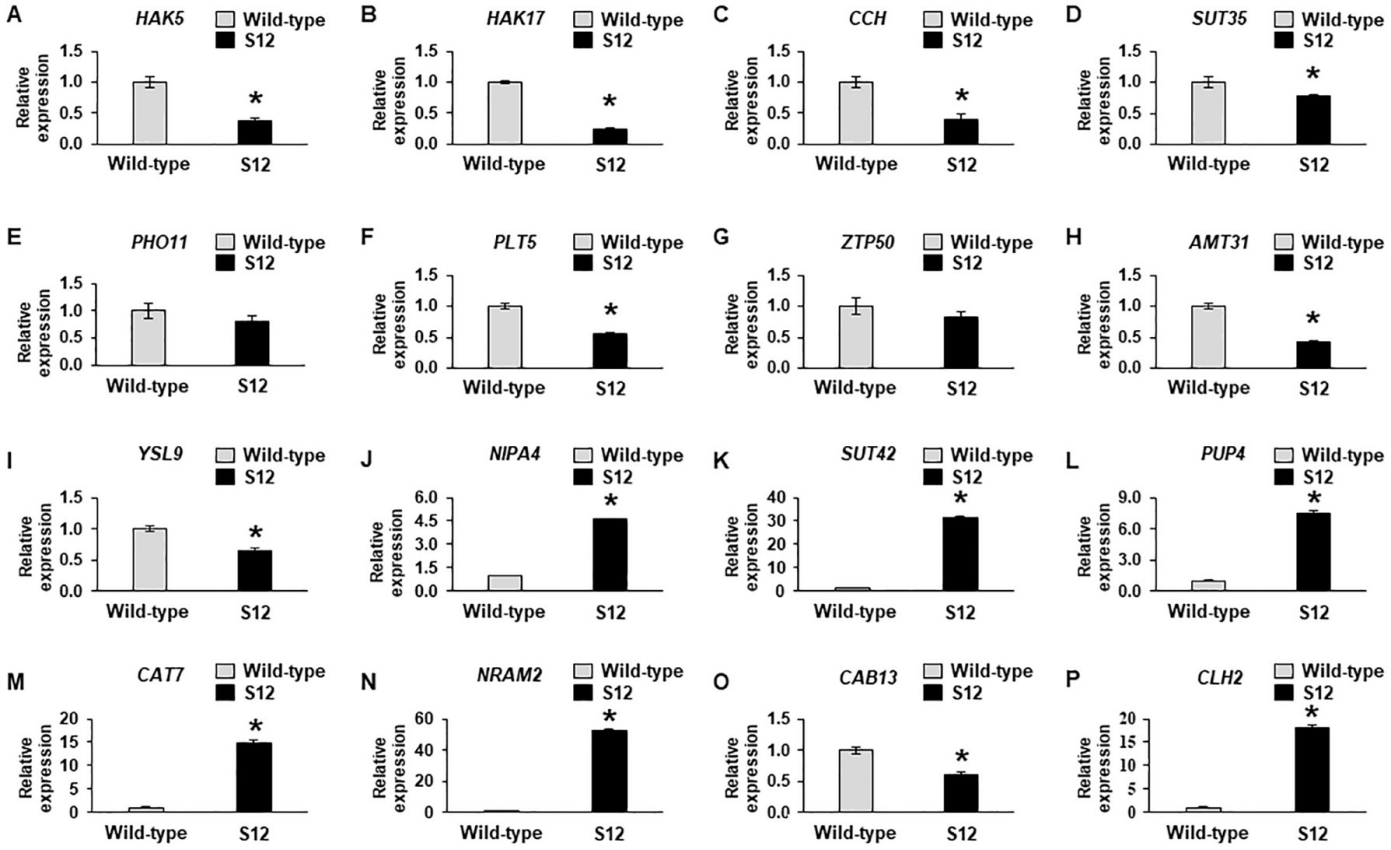
Quantitative real-time PCR analysis of 16 genes showing altered expression in the RNA-Seq analysis. The genes were associated with ion transporters (**A–N**), chlorophyll biosynthesis (**O**), and chlorophyll degradation (**P**). More specifically, **A–P** indicate the relative expression levels of Potassium transporter 5 (*HAK5*), Potassium transporter 17 (*HAK17*), Copper transport protein CCH (*CCH*), Sulfate transporter 3;5 (*SUT35*), Phosphate transporter PHO1 homolog 1 (*PHO11*), Polyol transporter (*PLT5*), Zinc transporter 50 (*ZTP50*), Ammonium transporter 3 member 1 (*AMT31*), Metal-nicotianamine transporter (*YSL9*), Magnesium transporter (*NIPA4*), Sulfate transporter 4;2 (*SUT42*), Amino acid permease family protein (*PUT4*), Cationic amino acid transporter 7 (*CAT7*), Metal transporter Nramp2 (*NRAM2*), Chlorophyll *a-b* binding protein 13 (*CAB13*), and Chlorophyllase-2 (*CLH2*), respectively. The *ACTIN* served as an internal control. Error bars indicate standard deviation (*n* = 3). The experiment was repeated three times with similar results.

We next identified genes associated with Chl metabolism to assay association between transcript and Chl levels. A previous study in the model plant Arabidopsis revealed that 16 genes are involved in the conversion of glutamyl-tRNA to Chl [47]. In addition, genes associated with several major Chl catabolites have been identified, including Chlorophyll *a*-*b* binding protein 13 (*CAB13*), non-yellow coloring 1 (*NYC1*), NYC1-like (*NOL*), chlorophyllases (*CLHs*), pheophorbide oxygenase (*PAO*), and red Chl catabolite reductase (*RCCR*) [48]. No significant differences in expression levels were observed between the WT and the S12 mutant for genes involved in Chl biosynthesis. However, elevated expression levels of *CLH2*, a gene involved in Chl degradation, was observed in the S12 mutant (Fig 9). To validate the RNA-Seq data, we used qRT-PCR to measure the expression levels of 16 genes, including genes associated with ion transport and Chl biosynthesis and degradation. The qRT-PCR analysis confirmed ~18 fold higher levels of *CLH2* in the S12 mutant. In comparison, *CAB13* and seven ion transporters, including two metal ion transporters, were down-regulated in the S12 mutants, by 1.65- and 1.3–4.05-fold, respectively (Fig 10O and 10A–10I). The expression levels of five other ion transporter genes were ~4.5–52.3-fold higher in the S12 mutants (Fig 10J–10N). Together, these results suggest that the reduced levels of Chl in the S12 mutant could be a result of increased Chl degradation and/or impaired ion transport associated with Chl biosynthesis.

## Discussion

In this study, we used the γ-ray-based mutagenesis procedure to isolate a leaf-color mutant in C*ymbidium*, which showed a notable decreases in Chl and carotenoid levels. The RNA-Seq analysis identified 2,267 DEGs, including 724 up-regulated and 529 down-regulated in the S12 mutant. A functional classification of these genes allowed us to identify the chlorophyllase gene, *CLH2*, which was induced ~18-fold in the S12 mutant. The *CLH2*-encoded enzyme facilitates Chl catabolism, and increased levels of *CLH2* have been associated with reduced Chl contents in a number of plants [49–56]. These results are consistent with a previous study [57], which suggested that the yellow-striped leaves of *C*. *sinense* variants were associated with increased Chl degradation. Notably, *C*. *sinense* variants characterized by Zhu et al. [57] showed ~1–3-fold higher expression levels of *CLH2* compared with the ~18-fold increase seen in the S12 mutant. Zhu et al. also observed a ~1–3-fold increase in the expression levels of *RCCR* [57], which acts downstream of *CLH2* in the Chl degradation pathway [57]. However, unlike the *C*. *sinense* variants characterized by Zhu et al. [57], the S12 mutant showed normal expression levels of *RCCR*. Consistent with previous studies [18, 23–26, 57], the S12 mutant also showed reduced levels of carotenoids, a phenotype commonly observed in Chl-deficient mutants. The S12 mutant also contained an altered Chl *a* to Chl *b* ratio, in which Chl *b* is required to stabilize the light-harvesting protein complex [58]. This, in turn, correlated with a reduced expression level of *NYC1* in the S12 mutant, which encodes a chlorophyll *b* reductase that catalyzes the conversion of Chl *b* to Chl *a*. Thus, a reduced Chl content and decreased Chl *a/b* ratio in the S12 mutant may be associated with fewer light-harvesting antenna complexes. In contrast, the S12 mutant showed normal expression levels of all 16 genes associated with Chl biosynthesis. This further suggests that the reduced Chl content in S12 leaves was likely associated with Chl catabolism and not biosynthesis.

Metals such as iron (Fe), copper (Cu), manganese (Mn), zinc (Zn), and magnesium (Mg) play key roles as cofactors in photosynthesis: Fe, as a cofactor for three photosynthetic electron transfer chain complexes; Cu, as a cofactor for thylakoid lumen electron transport protein plastocyanin; Mn, for photosystem II functions; Zn, plays a key role in the catalysis of chloroplastic ß-carbonic anhydrase enzyme; and Mg, in the center of the Chl ring [29, 45, 59]. The transitions of these metal ions are regulated to maintain cellular homeostasis, and excess metal ions cause oxidative stress because of their deleterious interactions with oxygen [29, 59]. In this study, we identified diverse metal ion transporters among down-regulated DEGs. In particular, the down-regulated expression levels of *CCH* (Cu-ion transporter) and *YSL9* (Fe-ion transporter) were confirmed by qRT-PCR (Table 5). The Arabidopsis *CCH* gene is a functional homolog of the yeast (*Saccharomyces cerevisiae*) gene Anti-oxidant 1, which, when mutated, results in a reduced Fe-uptake capability [60]. Senoura et al. [61] reported that OsYSL9 mainly localizes in the plasma membrane and transports Fe(II)-nicotianamine and Fe(III)-deoxymugineic acid. The expression of *OsYSL9* is repressed in the leaves under Fe-starvation conditions. The down-regulated expressions of *CCH* and *YSL9* could affect the Fe ion transition in the cell, which could be correlated with the reduced Chl content in the S12 mutant. NRAMP proteins are involved in Fe homeostasis [33], and the expression levels of *AtNRAMP1*, *3*, and *4* are up-regulated in response to Fe deficiency [62, 63]. Additionally, *AtNRAMP3* and *4* remobilize vacuolar Mn in leaves and have important roles in photosystem II functions [64]. In cyanobacteria, the ABC-type Mn transport complex is induced under Mn-starvation conditions [65]. In the S12 mutant, the reduced Fe content is expected because of the down-regulated expression levels of *CCH* and *YSL9* (Table 5). Additionally, the up-regulated expression levels of *NRAM2* and the ABC transporter family were identified (Table 4), which was consistent with previous reports [62–65].

In the present study, we found that seven genes involved in ion transport, including two metal ion transporters (*CCH* and *YSL9*), were down-regulated, and *CLH2*, associated with Chl degradation, was up-regulated in the yellow leaf-color mutant, S12. This provides useful information for understanding Chl biosynthesis and degradation in *Cymbidium*. In addition, these results show that γ-ray-based mutagenesis can be employed as a useful tool to generate genetic diversity among orchid species.

## Supporting information

S1 Table Primers used for qRT-PCR analysis. (DOCX)

S2 Table Functional classification of differentially expressed genes identified by KEGG clusters of orthologous genes (KOG) analysis. (XLS)
