# Supplementary material for: Transcriptome analysis to identify candidate genes associated with the yellow-leaf phenotype of a Cymbidium mutant generated by γ-irradiation
Source: PLoS One. 2020 Jan 29;15(1):e0228078. doi: 10.1371/journal.pone.0228078 (PMC6988911; doi:10.1371/journal.pone.0228078)
Supplement: S1 Table — (DOCX) [file pone.0228078.s001.docx]

**S1 Table. List of primers used for qRT-PCR analysis.**

| Trinity ID | Primer Name | Sequence |
| --- | --- | --- |
| TRINITY_DN35346_c0_g1 | HAK5-F | TTG CAA GTT GGG CTC TTT CT |
|  | HAK5-R | GAA CCA CAG CAC AAT GAT GG |
| TRINITY_DN86452_c2_g1 | HAK17-F | GCA ATT GCT TGG GGG TAC TA |
|  | HAK17-R | AAA TGG GCC AGT AGA CAT CG |
| TRINITY_DN82926_c0_g1 | CCH-F | CGT TTC CGA CCA CGT ATT CT |
|  | CCH-R | CCC TCT TAA CGG AAC CAA CA |
| TRINITY_DN82149_c4_g1 | SUT35-F | CAG TTC AAG GGC TTG CTA GG |
|  | SUT35-R | CGC AAA GGC AAT TAC TCC TC |
| TRINITY_DN81484_c2_g1 | PHO11-F2 | TTT GTG GAG AGG GGT GAG TC |
|  | PHO11-R2 | CTA ACG CCG AAC TCT CTT GG |
| TRINITY_DN82844_c0_g1 | ZTP50-F | ATT CCT TTG GCG AAG GTT CT |
|  | ZTP50-R | CTT TGT GGA AAG CAT CAG CA |
| TRINITY_DN55717_c0_g1 | AMT31-F2 | TAT AAG GGC GTG GAT GGT GT |
|  | AMT31-R2 | ACC AGC ATG AGA AGC AGG TT |
| TRINITY_DN82053_c3_g1 | YSL9-F | CTG TGG CGT CAG ATC TCA AA |
|  | YSL9-R | CGA GGA CTT TGG TCC ATG TT |
| TRINITY_DN86390_c5_g1 | PLT5-F | AAG TAG TTG CCG AGG AAG CA |
|  | PLT5-R | ACG TCG CTT ATG TCG AGG TC |
| TRINITY_DN59808_c0_g2 | CAB13-F | TCT GCC CAG ACA CCA TCA TA |
|  | CAB13-R | CTC CTT CGC TGA AGA TTT G |
| TRINITY_DN80260_c3_g15 | NYC1-F | TTT GCT GTG AAT GAG CTT GG |
|  | NYC1-R | GCC CCA TCC ATA TTG AAC AC |
| TRINITY_DN74882_c1_g1 | CLH2-F | GCC CTT GCT TGT CTC AAC TC |
|  | CLH2-R | GAA TCA GGT CCT GCA ATG GT |
| Genebank: GU181353 | ACTIN-F | AAT CCC AAG GCA AAC AGA |
|  | ACTIN-R | CCA TCA CCA GAA TCC AG |
